# Electron beam lithography with feedback using *in situ* self-developed resist

**DOI:** 10.1186/1556-276X-9-184

**Published:** 2014-04-16

**Authors:** Ripon Kumar Dey, Bo Cui

**Affiliations:** 1 Department of Electrical and Computer Engineering, Waterloo Institute for Nanotechnology (WIN), University of Waterloo, 200 University Ave. West, Waterloo, ON N2L 3G1, Canada

**Keywords:** Electron beam lithography, Self-developing resist, Nitrocellulose

## Abstract

Due to the lack of feedback, conventional electron beam lithography (EBL) is a ‘blind’ open-loop process where the exposed pattern is examined only after *ex situ* resist development, which is too late for any improvement. Here, we report that self-developing nitrocellulose resist, for which the pattern shows up right after exposure without *ex situ* development, can be used as *in situ* feedback on the e-beam distortion and enlargement. We first exposed identical test pattern in nitrocellulose at different locations within the writing field; then, we examined *in situ* at high magnification the exposed patterns and adjusted the beam (notably working distance) accordingly. The process was repeated until we achieved a relatively uniform shape/size distribution of the exposed pattern across the entire writing field. Once the beam was optimized using nitrocellulose resist, under the same optimal condition, we exposed the common resist PMMA. We achieved approximately 80-nm resolution across the entire writing field of 1 mm × 1 mm, as compared to 210 nm without the beam optimization process.

## Background

Electron beam lithography (EBL) is the most popular nanolithography method for research and device prototyping. Due to its much lower cost, most EBL systems for academic research are based on scanning electron microscope (SEM) without dynamic compensation. For such systems, the beam is typically optimized (stigmation compensated and well focused) at high magnification (e.g. ×100,000), so only the central spot of the writing field is optimized to attain a beam spot size of a few nanometers. At a distance farther away from the center, the beam spot is larger due to beam distortion and deterioration of focus. Due to the lack of *in situ* feedback, conventional EBL is a ‘blind’ open-loop process where the exposed pattern is examined only after *ex situ* resist development, which is too late for any improvement. Therefore, it is highly desirable to examine in situ the electron beam and optimize it before the time-consuming exposure of large-area pattern. This is particularly important for exposing large-area patterns that, in order to keep a reasonable exposure time, necessitates a large writing field and high beam current, which both magnify the issue of beam enlargement and distortion near the writing field corners. For instance, to expose a (1 cm)^2^ area with a writing field of (100 μm)^2^ using the Raith 150^TWO^ system (Dortmund, Germany), the total time for stage movement (10^4^ movements to expose the 10^4^ writing fields) would be 40,000 s (11 h) for a stage movement time between adjacent writing fields of 4 s. Obviously, the larger the pattern area is, the more significant the use of a large writing field is, though at the cost of reduced resolution. Furthermore, if all the structures for a device can be put inside one large writing field, the stitching error between the structures would be eliminated. Previously *in situ* feedback on electron beam drift based on imaging a mark or a grid pre-patterned on the substrate was reported [1-3], but no *in situ* feedback on electron beam spot size has been demonstrated.

Here, we propose to use self-developing resist, for which the exposed pattern shows up right upon exposure without an extra development step, as *in situ* feedback for the first time. With this closed-loop process, the beam spot can be optimized globally across an entire writing field, such that the beam spot size is evenly distributed. That is, the optimized beam spot size will be larger at the writing field center than obtained using conventional beam adjustment procedure, but much smaller near the writing field corners, thus allowing reasonably high-resolution patterning across the entire large writing field. Our procedure involves first writing test patterns in the self-developing resist at the center and corners within the writing field, analyzing them using SEM at high magnification, adjusting the electron beam (notably working distance and stigmation), and repeating the task until achieving a relatively uniform shape/size distribution across the entire writing field. Afterwards, under the same optimized beam condition, the exposure will be carried out to pattern the device using normal high-performance resist like PMMA. It is noted that here *in situ* optimization is important as otherwise the electron column condition would be different if one has to turn off the system to take out the exposed sample for *ex situ* development to examine the beam spot size at different locations. Obviously, the same self-developing resist can also be used as *in situ* feedback for optimizing writing field alignment to minimize the stitching error between adjacent fields, and we have reproducibly achieved nearly perfect (<50-nm stitching error) alignment with a large writing field of 1 mm × 1 mm [4].

The *in situ* feedback is provided by self-developing resist, for which the exposed test pattern shows up and can be examined right after exposure by SEM at high magnification. This is in contrast to conventional resist that requires *ex situ* development using solvent or aqueous developer. Self-developing electron or ion beam resists had been extensively studied in the 1980s. For instance, metal halides such as AlF_3_ are decomposed to form volatile fluorine gas upon electron beam exposure; thus, they behave as a positive self-developing resist [5-9]. Similarly, nitrocellulose is decomposed upon exposure to electron or ion beam; thus, it is also a positive self-developing resist [10-13]. However, those self-developing resists are nearly forgotten by the EBL community after their discovery. We believe this is because the metal halide resists suffer from extremely low sensitivity and inability to expose arbitrary structure other than very thin line and dot patterns since the decomposition product metallic Al cannot migrate far away from the directly exposed area, whereas nitrocellulose resist always leave behind a thick non-volatile residual layer. In fact, nitrocellulose was mostly used as an ion beam resist for which the residual layer is thinner because physical bombardment by ion beam can help remove the non-volatile species [14]. Though metal halides offer extremely high resolution, the film is found to be degraded by humidity after long (several weeks) exposure to air. More recently, ice and frozen carbon dioxide were shown to behave as an electron beam resist without the need of a development step [15-18]. However, they both require significant modification of the EBL system to maintain a low temperature, which greatly limits their application. Lastly, PMMA and ZEP resist have also demonstrated self-developing behavior, yet the resist thickness reduction due to over-exposure at approximately 15 times normal clearance dose was less than 30% of the original film thickness if without *ex situ* post-exposure thermal annealing [19]. Therefore, here, we have chosen nitrocellulose for the purpose of *in situ* feedback. As expected, it behaves like a positive resist since e-beam exposure can also generate secondary electrons to decompose the resist, as ion beam does, and the amount of residual layer is significant. However, a thick residual layer, though undesirable since it lowers SEM imaging contrast, is acceptable for the purpose of *in situ* feedback. Interestingly, nitrocellulose was also found to be developable using a solvent developer to give a mixed positive and negative tone behavior.

## Methods

As-purchased nitrocellulose (Sigma-Aldrich, St. Louis, MO, USA) was further diluted with pentyl acetate at 1:1 volume ratio, which gave a film thickness of 300 nm by spin coating. The film was then baked at 80°C for 5 min to drive away the solvent. To obtain the contrast curve of the nitrocellulose resist, we exposed an array of large squares each with 5 μm × 5 μm at 20 keV with exponentially increasing doses using a Raith 150^TWO^ electron beam lithography system. As a self-developing resist, nitrocellulose displays a positive tone right after exposure. It is also interesting to investigate whether the exposed resist can be developed using a solvent, for which we tried to develop the resist using pentyl acetate and observed a mixture of positive and negative tone behavior. The contrast curves with and without solvent development were measured using atomic force microscope (AFM), with the film thickness measured by Dektak profilometer (Veeco Instruments Inc., Plainview, NY, USA). For the case with solvent development, the development time was long enough to remove all the resist in the unexposed area. In the contrast curves, the remaining resist thickness was normalized to the film thickness after spin coating and baking. In order to investigate the high resolution capability of nitrocellulose resist, periodic line array with a period of 600 nm was exposed at 20 keV over a broad line dose range and subsequently coated with 30 nm Cr for SEM imaging.

For electron beam optimization across a large writing field, we first followed the standard process to adjust the beam at a high magnification of × 50,000. Then, we exposed, with exponentially increasing line doses of 30 to 500 nC/cm for nitrocellulose, the test pattern containing five identical designs at the writing field center and four corners, respectively. Here, a large writing field of 1 mm × 1 mm obtained at a low magnification of × 100 was chosen. Afterwards, we examined the exposed pattern at high magnification, which naturally revealed a well-defined structure at the writing field center but poorly defined ones at the corners. This is because, when the center is well focused, the corners are actually greatly defocused because the distance from the electron objective lens to the corner is longer than to the center. Next, the same procedure was repeated at a new location, but with an increased working distance value (the working distance value was entered manually, without physically raising or lowering the stage). At this increased working distance value, the writing field center will be defocused, but the corners will become less defocused. In principle, the stigmation values can also be finely tuned, but here we focus our effort on optimizing the working distance. After several iterations, similar exposed line widths were observed at the writing field center and corners, which suggests that an optimal working distance was achieved to give a relatively uniform exposed pattern across the entire writing field. To verify the effectiveness of our method, under the optimal exposure parameters, we exposed the high-resolution resist PMMA (100-nm thickness, coated on silicon that was mounted beside the wafer coated with nitrocellulose) at line dose of 400 to 3,300 pC/cm. Note that the optimal exposure parameters remain valid as long as the aperture size (that determines the depth of focus as well as beam current) and working distance remain the same (if the sample is at a height level different from the nitrocellulose film, the stage can be raised/lowered to obtain roughly the same working distance). After development using the standard developer MIBK:IPA (1:3) for 40 s, the pattern was coated with 10-nm Cr and examined by SEM.

## Results and discussion

### Exposure properties of nitrocellulose with and without *ex situ* solvent development

**Figure 1:**
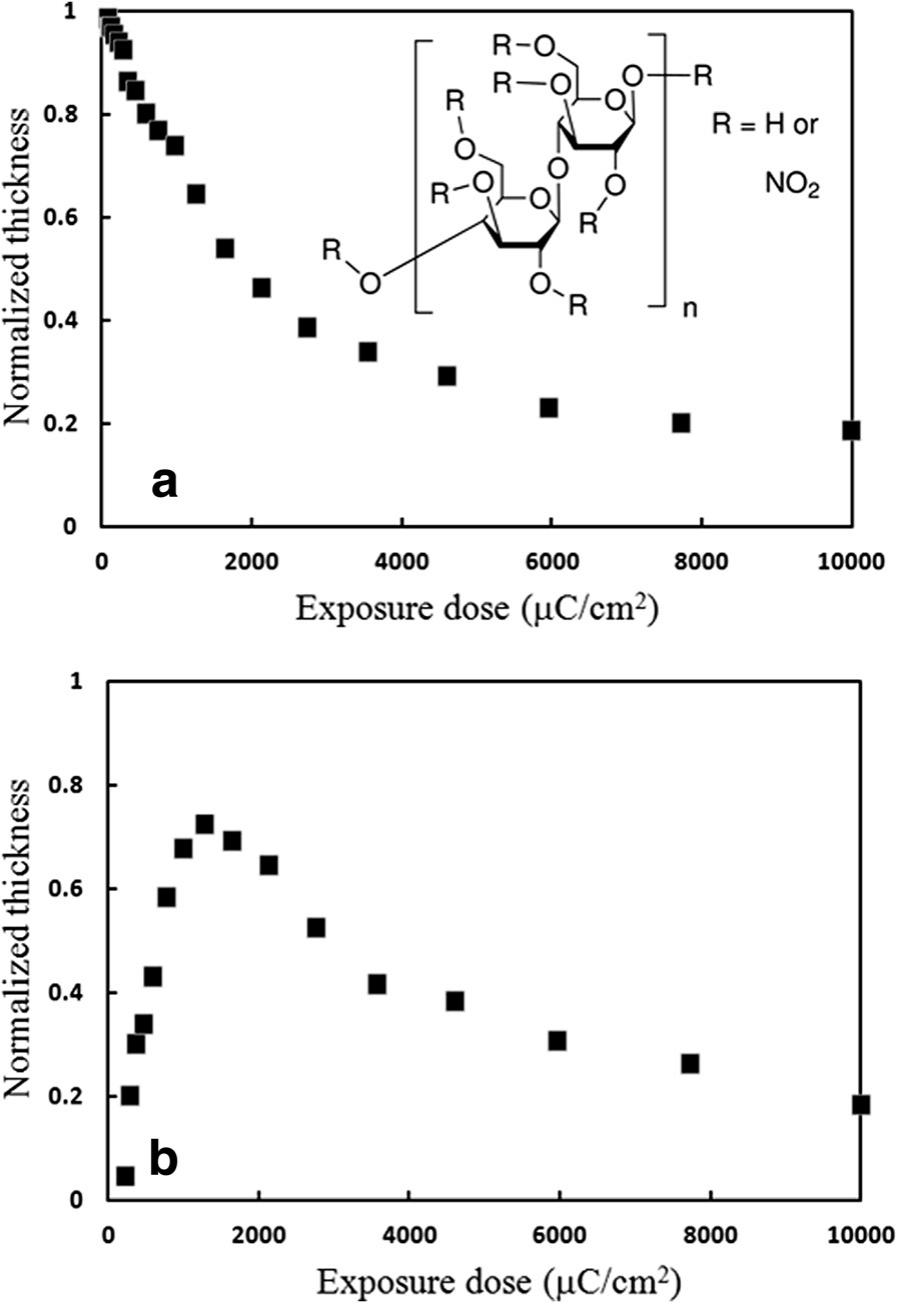
**Contrast curves for nitrocellulose.** Exposure at 20 keV without *ex situ* development **(a)** and with 60-s development in pentyl acetate **(b)**. The inset in **(a)** shows the chemical structure of nitrocellulose.

**Figure 2:**
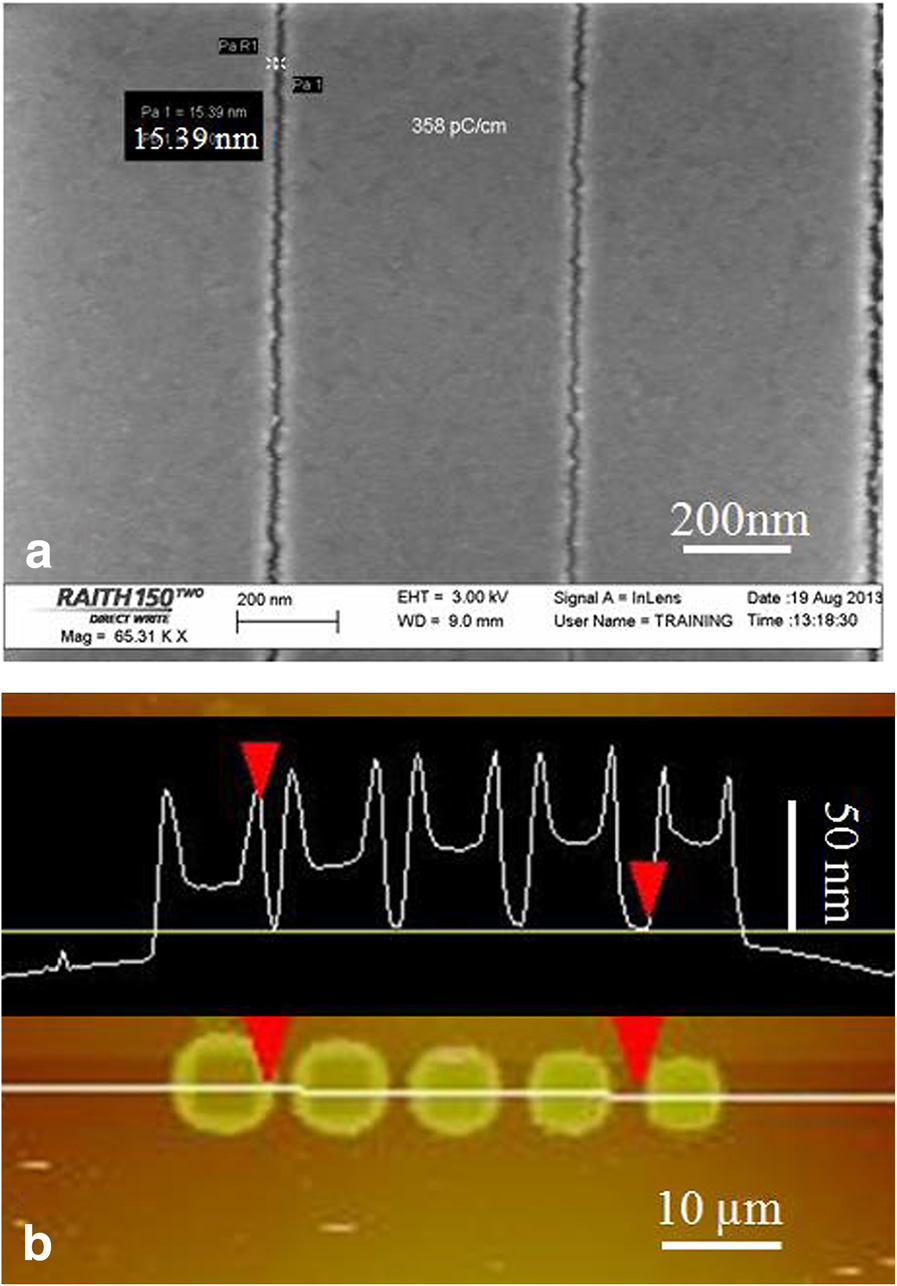
**SEM and AFM images of structures in nitrocellulose. (a)** SEM image of line array exposed in nitrocellulose without *ex situ* development, showing a line width of 15 nm. **(b)** AFM image and cross-section of complex microstructure exposed in nitrocellulose after *ex situ* solvent development.

Figure 1 shows the contrast curves for nitrocellulose exposed at 20 keV without *ex situ* development (Figure 1a) and with pentyl acetate development for 60 s (Figure 1b). As expected, for both cases, a thick residual layer of nearly approximately 20% of the original film thickness was left behind even at very high exposure doses. Consequently, nitrocellulose is not a useful electron beam resist for pattern transfer purpose, but it is acceptable for the purpose of providing *in situ* feedback for electron beam lithography. As a self-developing resist, the sensitivity (defined as the dose for 50% remaining thickness) is about 2,000 μC/cm^2^. The sensitivity is about 10 times lower than PMMA (clearing dose approximately 200 μC/cm^2^ at 20 keV), but again this is not a serious drawback for our purpose since the time to expose the test pattern is short enough. As for the contrast, one cannot derive a meaningful value from the contrast curve, yet clearly the nitrocellulose resist has a low contrast, which makes it unsuitable for exposing high-resolution dense pattern. Nonetheless, it is capable of delineating high-resolution sparse pattern for which proximity effect is insignificant, as seen in Figure 2a that shows a resolution down to 15 nm. Actually, another very low contrast resist SU-8 has also achieved a high resolution of 24 nm [20].

After additional solvent development, the contrast curve (Figure 1b) shows a mixed behavior, rather than a simple positive or negative tone behavior. At very low exposure doses, since the unexposed resist is soluble in pentyl acetate developer whereas electron beam exposure decomposed the resist to generate less soluble decomposition product, the resist exhibited a negative tone. At higher doses, on the one hand, the resist was increasingly decomposed and vaporized with increasing doses, which led to the tendency of positive tone; on the other hand, as the degree of decomposition increased, the decomposition product became less soluble in the solvent developer, resulting in the tendency of negative tone after solvent development. As a consequence of those two competing trends, there exists a turning point exposure dose (approximately 1,200 μC/cm^2^) that gave a maximum remaining thickness. Such an exposure behavior can lead to complex structure as shown in Figure 2b, which is due to proximity exposure at the surrounding area beyond the directly exposed area. In fact, such kind of mixed exposure property is well known for a long time for PMMA that displays a positive tone at low doses and becomes a negative tone at approximately 10 times higher doses [21], which was also employed to generate complex structures [22]. Though less known, another popular resist ZEP-520A actually also exhibits a mixed tone behavior just like PMMA [23]. However, unlike PMMA and ZEP for which the negative tone behavior appears only after roughly 10 times higher doses, for nitrocellulose, the negative tone behavior proceeds the positive tone, and the dose ranges for the two tones have a large overlap and thus they are not clearly separated.

### E-beam working distance optimization using nitrocellulose resist

**Figure 3:**
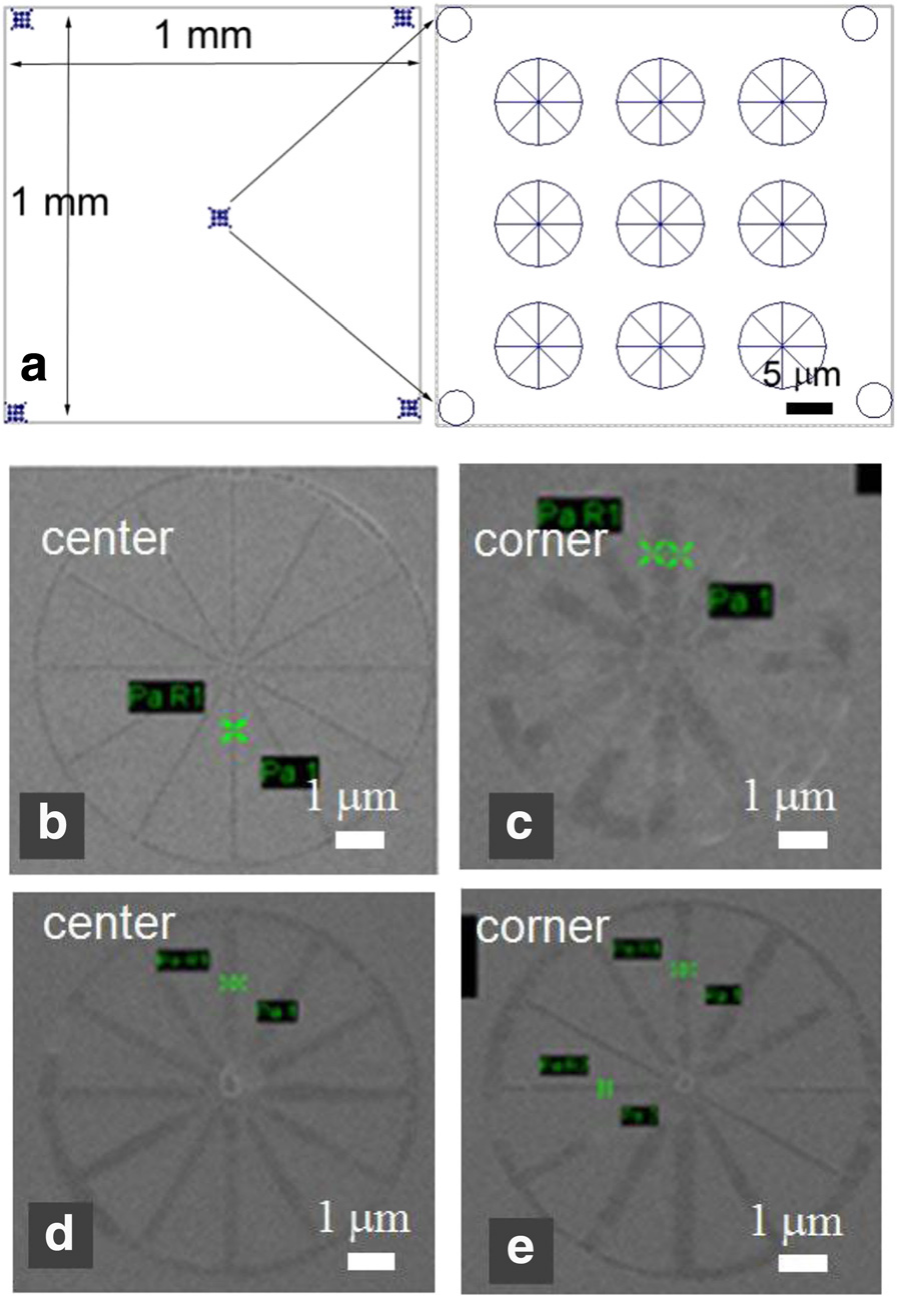
**CAD pattern design and structures exposed in nitrocellulose. (a)** The CAD pattern design consisting of five identical wheel array structures (see right side for zoom-in view) at the 1 mm × 1 mm writing field center and four corners. One wheel structure exposed in nitrocellulose at the center **(b)** and corner **(c)** without beam optimization by defocus. One wheel structure at the center **(d)** and corner **(e)** with beam optimization by defocusing at 37 μm.

Figure 3a illustrates the pattern design within the 1 mm × 1 mm writing field that consists of five identical wheel-structure array at the center and four corners, respectively, with the inset showing the wheel-structure array having exponentially increasing line doses from the upper left to the lower right wheel. A broad range of exposure dose is critical because a relatively low dose is needed to reveal the high resolution capability when the beam is well focused, yet a high dose is essential to self-develop the resist to a certain visible depth when the beam is seriously enlarged. The wheel design is advantageous as it contains lines along various directions, which ensures that some lines (those roughly along the beam spot elongation direction when there is severe astigmatism) would be adequately self-developed to become visible under SEM.

Figure 3b,c shows two wheel structures at the center and corner, respectively, when the electron beam was well focused at the writing field center with a working distance of 8 mm. As expected, the center wheel (50-nm-wide line at a dose of 34 nC/cm) was well defined, whereas the corner one (315-nm-wide line at a dose of 34 nC/cm, developed to a small depth) was seriously blurred. Here, the SEM image has a low contrast, which is because of the low yield of secondary electrons for the polymer resist at 20 kV (the imaging acceleration voltage has to be the same as the exposure voltage in order to maintain a consistent electron column condition). The contrast could be improved by coating the resist with a thin metal island film that allows vaporization of the decomposed resist through the island film. After several iterations with increasing working distance values, we achieved relatively uniform pattern definition at a defocus value of 37 μm (i.e., working distance 8.037 mm), as shown in Figure 3d,e for the two wheel structures at the center and corner, respectively. As a simple estimation, the distance from the electron object lens to the writing field center is 8 mm, whereas that from the lens to the writing field corner is (8^2^ + 0.5^2^ + 0.5^2^)^1/2^ = 8.031 mm or 31 μm farther than to the writing field center, which is in the same order as our optimal defocus value. Clearly, the optimal defocus value and the degree of improvement using our method depend on the depth of focus, which is inversely proportional to the aperture size and proportional to the working distance. Our approach would be less effective when the depth of focus is high that leads to less beam broadening and distortion at writing field corners. However, high depth of focus means either the aperture size is small that results in long exposure time because beam current is roughly proportional to the square of aperture size, and/or the working distance is large that makes the exposure more susceptible to electromagnetic and vibrational noise.

**Figure 4:**
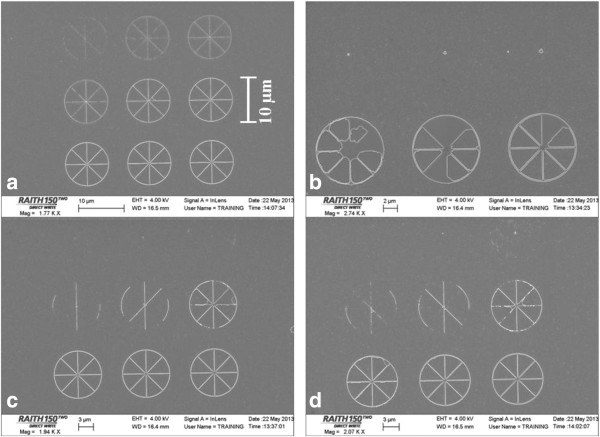
**Cr pattern created by electron beam lithography with PMMA resist followed by a liftoff process.** Wheel array at writing field center **(a)** and corner **(b)** exposed without beam optimization by defocus. Wheel array at writing field center **(c)** and corner **(d)** exposed with beam optimization using self-developing nitrocellulose resist. The exposure dose increases from the top left to the lower right wheel structure.

To verify the optimal beam adjustment, under the same exposure condition with and without a defocus of 37 μm, we exposed PMMA at a dose range appropriate for PMMA and carried out a standard liftoff process of 10-nm Cr. Figure 4 shows the resulting wheel array pattern in Cr. The Cr line widths at different doses and positions within the writing field, with and without beam optimization by defocusing, are listed in Table 1. When the dose is low and/or the beam is greatly broadened, the resist was not developed to the bottom, leading to no pattern after Cr liftoff. As expected, without defocus, the minimum line width at the writing field corner is 210 nm; with optimal defocus, fairly high resolution of about 80 nm was obtained across the entire writing field Lastly, nitrocellulose resist can also be used as *in situ* feedback for optimizing writing field alignment to minimize the stitching error between adjacent fields [4]. By exposing periodic test patterns in nitrocellulose at the writing field boundaries and viewing them at high magnification, the magnitude of the stitching error can be measured precisely, which can be used to derive the optimal zoom and rotation value in the Raith 150^TWO^ system. We have reproducibly obtained nearly perfect (<50-nm stitching error) alignment with a large writing field of 1 mm × 1 mm, as compared to an average stitching error of approximately 500 nm obtained without using nitrocellulose as *in situ* feedback.

**Table 1:** The resulting Cr line width as a function of exposure dose with or without beam optimization

**Line dose (nC/cm)**	**Well focused at the center (nm)**	**Well focused at the corner (nm)**	**Defocused at the center (nm)**	**Defocused at the corner (nm)**
0.4	42	Resist not developed to the bottom due to beam broadening at the writing field corner, thus no Cr pattern after liftoff	Resist developed to the bottom	Resist not developed to the bottom
0.56	43			
0.79	47			
1.10	51		78	84
1.15	62		89	91
2.15	70		120	128
3.01	91	210	127	138
4.21	108	251	146	152
5.90	117	272	167	172

## Conclusions

Here, we studied the exposure properties of nitrocellulose resist and its application as *in situ* feedback for electron beam optimization in electron beam lithography. It was found that, as a self-developing resist, nitrocellulose showed low sensitivity and low contrast, making it unsuitable for patterning high-resolution dense features. Nevertheless, it achieved 15-nm resolution for sparse pattern where proximity effect is insignificant. In addition to self-development, nitrocellulose resist can also be developed using a solvent that displayed a mixed tone behavior - negative tone for low doses and positive for high doses. Using nitrocellulose as *in situ* feedback to optimize the electron beam (notably working distance) across a large writing field of 1 mm × 1 mm, we achieved approximately 80-nm resolution across the entire writing field, as compared to 210 nm (occurred at the writing field corners) without the beam optimization process. This approach is most efficient in reducing the writing time for large writing field size such as 1 mm × 1 mm as needed for large area exposure of moderate resolution pattern.

## Competing interests

The authors declare that they have no competing interests.

## Authors’ contributions

RD carried out the experiment and drafted the manuscript. BC designed the experiment and revised the manuscript. Both authors read and approved the final manuscript.
